# Construction of SnO_2_−Graphene Composite with Half-Supported Cluster Structure as Anode toward Superior Lithium Storage Properties

**DOI:** 10.1038/s41598-017-03603-1

**Published:** 2017-06-12

**Authors:** Chengling Zhu, Zhixin Chen, Shenmin Zhu, Yao Li, Hui Pan, Xin Meng, Muhammad Imtiaz, Di Zhang

**Affiliations:** 1 State Key Laboratory of Metal Matrix Composites, Shanghai Jiao Tong University, Shanghai, 200240 P.R. China; 2 School of Mechanical, Materials & Mechatronics Engineering, University of Wollongong, Wollongong, NSW 2522 Australia; 3 National Engineering Research Center for Nanotechnology, Shanghai, P.R. China

## Abstract

Inspired by nature, herein we designed a novel construction of SnO_2_ anodes with an extremely high lithium storage performance. By utilizing small sheets of graphene oxide, the partitioned-pomegranate-like structure was constructed (SnO_2_@C@half-rGO), in which the porous clusters of SnO_2_ nanoparticles are partially supported by reduced graphene oxide sheets while the rest part is exposed (half-supported), like partitioned pomegranates. When served as anode for lithium-ion batteries, SnO_2_@C@half-rGO exhibited considerably high specific capacity (1034.5 mAh g^−1^ after 200 cycles at 100 mA g^−1^), superior rate performance and remarkable durability (370.3 mAh g^−1^ after 10000 cycles at 5 A g^−1^). When coupled with graphitized porous carbon cathode for lithium-ion hybrid capacitors, the fabricated devices delivered a high energy density of 257 Wh kg^−1^ at ∼200 W kg^−1^ and maintained 79 Wh kg^−1^ at a super-high power density of ∼20 kW kg^−1^ within a wide voltage window up to 4 V. This facile and scalable approach demonstrates a new architecture for graphene-based composite for practical use in energy storage with high performance.

## Introduction

With the rapid evolution of new automobile propulsion and the continuous lightening of electronic devices, the development of lithium-ion batteries (LIBs) toward high energy density, high power density, and long cycling life has been put under urgent. As one of the most important issues in LIBs, new generations of anode materials have been explored, aimed to transcend the theoretical limit of conventional graphite anodes (372 mAh g^−1^). Besides the novel nanostructured carbon materials such as multimodal porous carbon^1, 2^, hollow core carbon spheres^3^, and mesoporous carbon nanofibers^4^, two kinds of substances are of great interest for their high theoretical capacities: one kind being reversibly lithiated/delithiated through conversion reaction (Fe_2_O_3_, Fe_3_O_4_, MnO_2_, CoO, *etc*.), and the other through alloying reaction (Si, Ge, Sn, SnO_2_, *etc*.)^5–12^. Unfortunately, the pulverization and loss of electrical contact caused by the significant volume change during lithiation/delithiation reaction have been identified as main reasons for the capacity fading of these new anodes^10, 11, 13, 14^.

Graphene, mostly reduced graphene oxide (rGO), has been widely used to overcome the flaw of various new-type anode materials^6–9, 15–17^. The ultimate electrical conductivity, ultrahigh specific surface area, and unique two-dimensional shape of graphene exert substantial effects on both the physicochemical property and the nanostructure of these anodes. Packaging nanoparticles of anode materials into clusters and encapsulating the clusters with rGO sheets has been demonstrated as an effective approach for these materials such as Fe_3_O_4_, ZnMn_2_O_4_ and SnO_2_^6, 12, 18^. Wang *et al*. prepared ZnMn_2_O_4_ porous spheres and encapsulated them with GO^18^. The composite anode exhibited a capacity of 926.4 mAh g^−1^ at current density of 200 mA g^−1^. It is believed that the electrical conductivity of the composite electrode can be improved by rGO, and the voids between the nanoparticles can buffer volume change and prevent agglomeration and pulverization. Unfortunately, when the ZnMn_2_O_4_/rGO anode was tested at 1 A g^−1^, a decline tendency was found only after 120 cycles. The reason may lie in the fact that the encapsulated rGO shell would hinder the rapid penetration and transfer of lithium ions (Li^+^)^19, 20^. It is still a great challenge to design novel structures of graphene-based composite anodes to afford high transfer speed of Li^+^ as well as high reversible capacity and stability, which also accords with the purpose of applying these new-type anode materials in lithium-ion hybrid capacitors (LIHCs) to get energy storage devices with high power density^21^.

In fact, nature provides us inspiration for the design of new materials. Learning from nature, Cui *et al*. designed a pomegranate-like anode, where single-phased silicon nanoparticles are encapsulated by a conductive carbon layer that leaves enough room for expansion and contraction during charge-discharge process. The anode showed an excellent cycle life (97% capacity retention after 1000 cycles), high coulombic efficiency (99.87%) and volumetric capacity (1270 mAh cm^−3^)^22^. The naturally formed structure of pomegranates is an impressed model in the design of anode materials to accommodate a large volume expansion. Taking advantage of the unique properties of graphene together with the fantastic pomegranate structure, we recently synthesized an anode material based on SnO_2_, where mesoporous SnO_2_ clusters were encapsulated by amorphous carbon layers and then wrapped with rGO sheets. The obtained materials exhibited a high reversible capacity of 924 mAh g^−1^ at 100 mA g^−1^. However, the encapsulated dual thick carbon shell hindered the rapid penetration and transfer of Li^+^ and resulted in an unsatisfactory rate performance (240 mAh g^−1^ at 3 A g^−1^)^12^.

**Figure 1:**
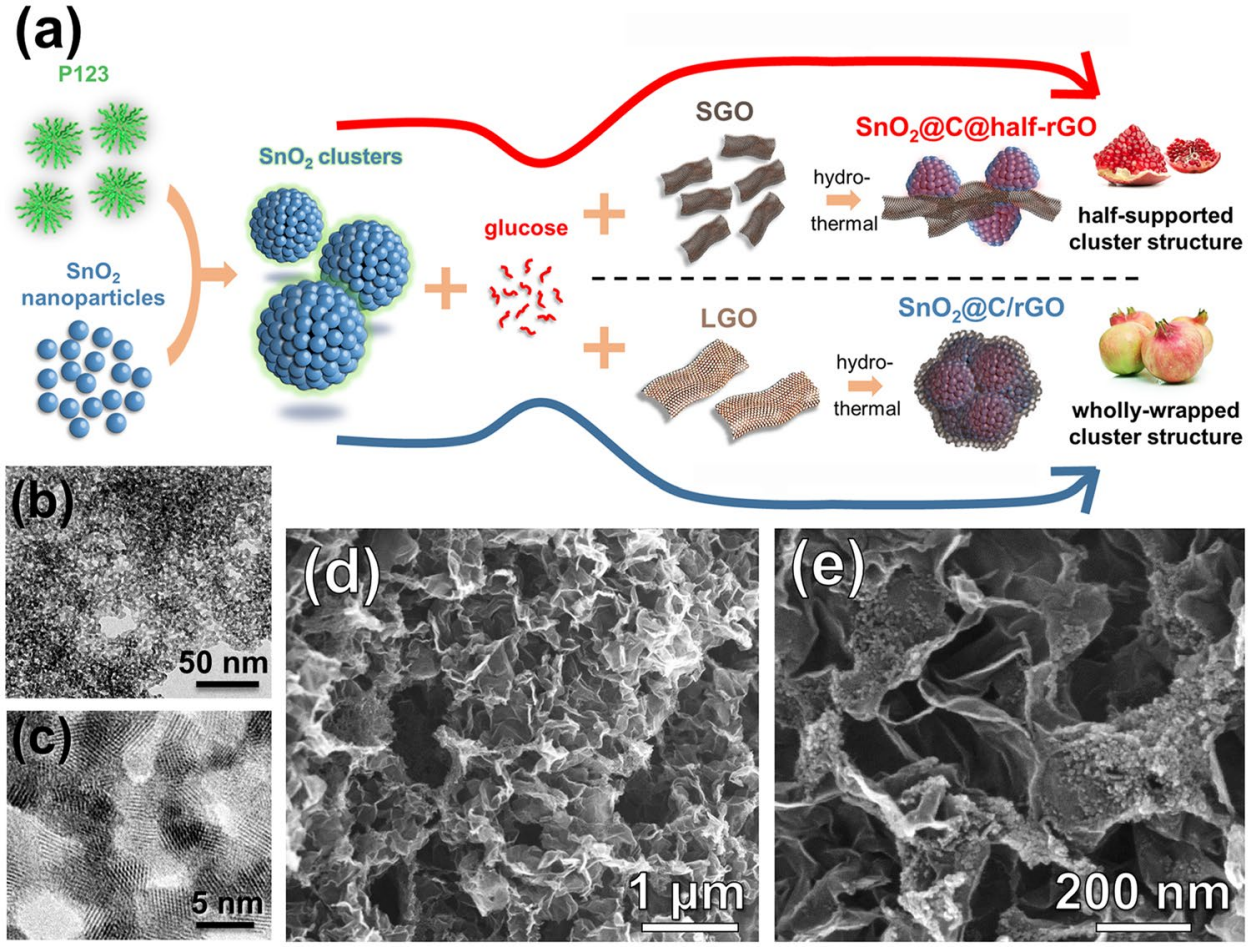
(**a**)Graphical synthesis route of SnO_2_@C@half-rGO and SnO_2_@C/rGO. (**b,c**) TEM images of the SnO_2_ nanoparticles. (**d,e**) SEM images of SnO_2_@C@half-rGO.

Imitating the structure of partitioned pomegranates, herein we pioneered a brand new structure for extremely high-performance SnO_2_-rGO composite anode, which can be applied in both LIBs and LIHCs (Fig. 1a). Small sheets of graphene oxide (SGO) were used to construct this delicate structure of SnO_2_ anodes (SnO_2_@C@half-rGO), in which the porous clusters of SnO_2_ nanoparticles are partially supported by rGO sheets and partially exposed. The SnO_2_ nanoparticles inside the porous clusters are individually covered by thin amorphous carbon shells. The structure in whole is just like partitioned pomegranates. Such a design has multiple advantages: (1) the thin amorphous carbon shell not only limits most SEI formation to the outer surface, but also enables the fast diffusion of Li^+^; the void space inside the clusters is well defined and evenly distributed around each nanoparticle, which can buffer the expansion of the nanoparticles; (2) rGO provides a conducting framework. Compared with the wholly-wrapped control sample (SnO_2_@C/rGO, Fig. 1a) prepared using large sheets of graphene oxide (LGO), SnO_2_@C@half-rGO can maintain the conductive matrix of rGO while open the rapid access for Li^+^.

As the result, SnO_2_@C@half-rGO gained a much improved performance as the anode for LIBs. It showed a high reversible capacity up to 1034.5 mAh g^−1^ at 100 mA g^−1^ and superior rate performance and stability (370.3 mAh g^−1^ after 10000 cycles at 5 A g^−1^). When coupled with graphitized porous carbon (gpC) cathode into LIHCs, the obtained SnO_2_@C@half-rGO//gpC device exhibited a considerably high energy density of 257 Wh kg^−1^ at ∼200 W kg^−1^, and 79 Wh kg^−1^ was maintained at a super-high power density of ∼20 kW kg^−1^.

## Results

The synthesis route of SnO_2_@C@half-rGO is schematically represented in Fig. 1a. Firstly, the SnO_2_ nanoparticles were prepared as hydrosol. TEM images (Fig. 1b,c) of the SnO_2_ nanoparticles in hydrosol show a well-dispersed morphology, with a uniform size of ∼3 nm. The block copolymer surfactant P123 was mixed with the SnO_2_ hydrosol to gather the nanoparticles with ample amphiphilic groups, followed by the addition of glucose, which acted as both a reductant^23^ and the precursor of amorphous carbon. Subsequent introduction of small sheet GO (denoted as SGO) into the system followed by a hydrothermal process led to the reduction of SGO and the figuration of the target structure (SnO_2_@C@half-rGO).

The wrinkled and porous network of rGO in SnO_2_@C@half-rGO is manifested in the SEM image (Fig. 1d), while the nested secondary structure is shown in the magnified one (Fig. 1e). It can be clearly identified that the well-formed SnO_2_ clusters are supported by rGO sheets on part of the cluster surface (half-supported, for short). The element mapping images of Sn, C, and O (Supplementary Fig. S1) indicate a uniform distribution of SnO_2_ clusters on the rGO sheets in large scale.

**Figure 2:**
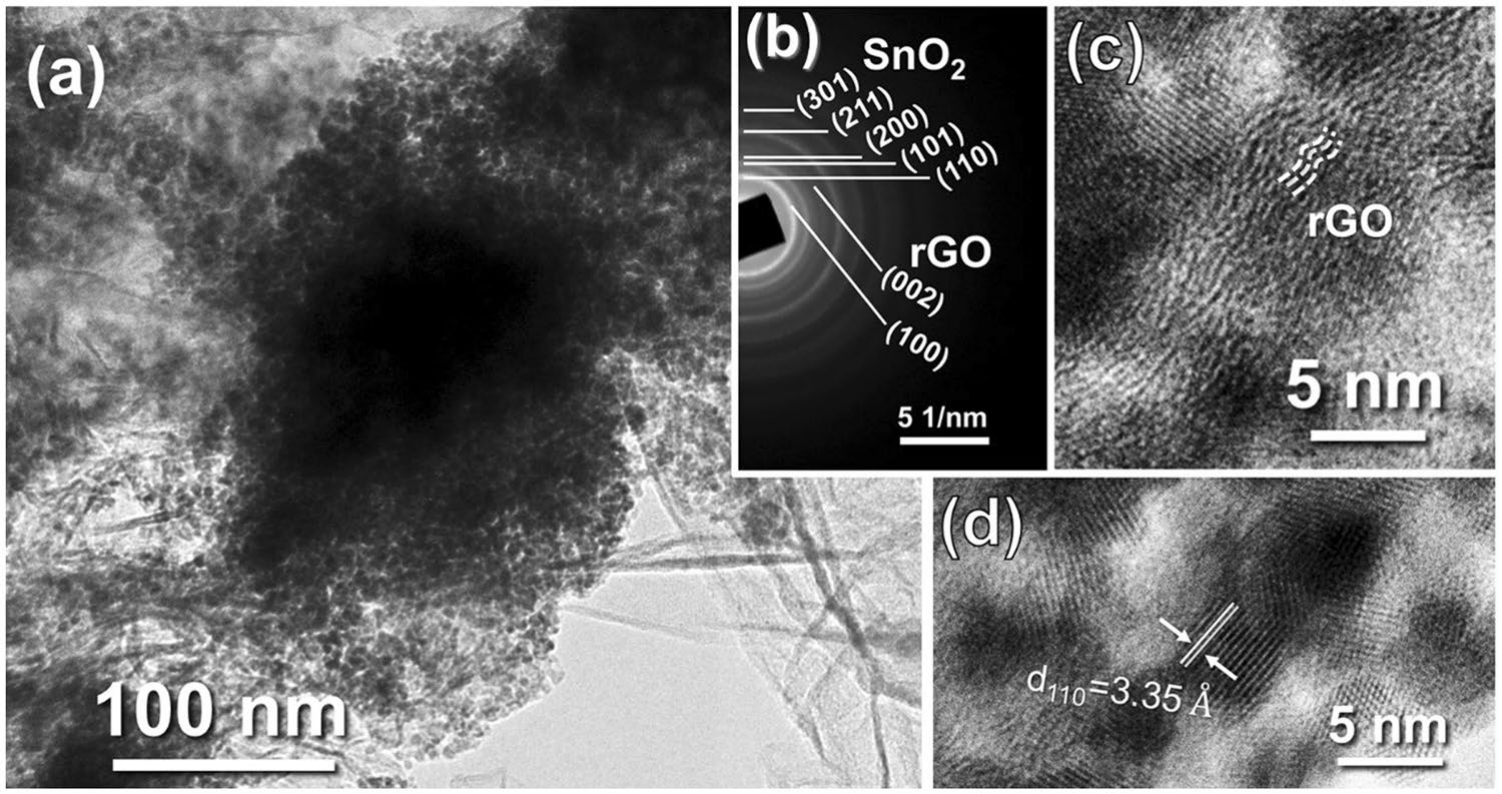
TEM image of SnO_2_@C@half-rGO. (**a**) The rGO-half-supported clusters in SnO_2_@C@half-rGO. (**b**) The SAED pattern of SnO_2_@C@half-rGO. (**c**) Magnified image on the edge of the clusters, showing the flexuose stripe of rGO lattice. (**d**) HRTEM of the SnO_2_ nanoparticles in SnO_2_@C@half-rGO.

**Figure 3:**
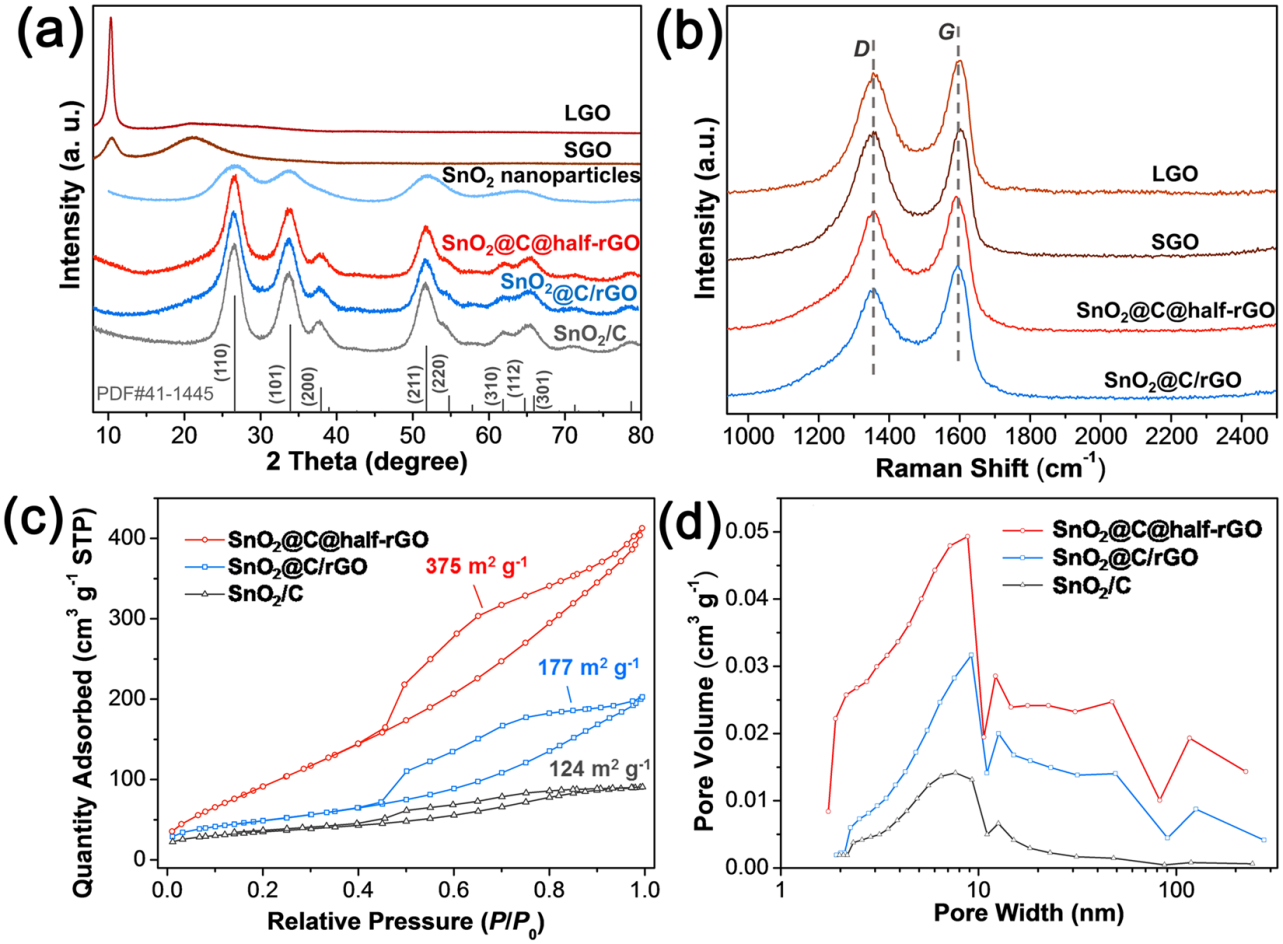
(**a**) XRD patterns of LGO, SGO, SnO_2_ nanoparticles, SnO_2_@C@half-rGO, SnO_2_@C/rGO, and SnO_2_/C. The standard peak position and intensity of PDF#41-1445 SnO_2_ are shown as verticle lines. (**b**) Raman spectra of LGO, SGO, SnO_2_@C@half-rGO, and SnO_2_@C/rGO. (**c**) Nitrogen adsorption/desorption isotherms and (**d**) pore size distribution of SnO_2_@C@half-rGO, SnO_2_@C/rGO and SnO_2_/C.

The detailed cluster structure of the SnO_2_ nanoparticles in TEM is clearly shown in Fig. 2a, while the void space spreads among the SnO_2_ particles. The diffraction rings rather than spots are observed on the SAED pattern (Fig. 2b), which is consistent with the ultra-small size of the SnO_2_ determined by XRD pattern (Fig. 3a). Moreover, two rings of the (100) and (002) lattice planes of rGO can be found, well corresponding to the flexuose stripe fingerprint of graphitic carbon in the HRTEM image (Fig. 2c). The rGO sheets are found to adhere tightly to the SnO_2_ clusters. In the magnified area of the SnO_2_ clusters (Fig. 2d), the lattice fringes of SnO_2_ can be captured more clearly, while amorphous substance is observed all around the SnO_2_ nanoparticles, which is inferred to be the amorphous carbon derived from glucose.

GO has long been applied as structure framework and conductivity reinforcement in various composites with nanoarchitectonic design for various application^24^. Generally, GO with micron-scale size was adopted. In this research, the use of SGO (with average lateral size of 0.42 μm) is a key point to construct this partitioned-pomegranate-like composite. The difference in the size and chemical properties of SGO and large sheet GO (named as LGO, the detailed information supplied in Supplementary Figs S2 and S3) can result in different nanostructures when they are introduced to fabricate composites with nanoparticles. While SGO can lead to a half-supported structure in SnO_2_@C@half-rGO, LGO can and will wholly wrap the SnO_2_ clusters, which was clearly observed in SEM and TEM (Supplementary Fig. S4).

The wholly-wrapped SnO_2_ clusters was abbreviated as SnO_2_@C/rGO and studied as a comparison sample (shown in Fig. 1a). In addition, another control sample was prepared with only SnO_2_ nanoparticles and amorphous carbon without GO, which was named SnO_2_/C (Supplementary Fig. S5).

The cassiterite phase of SnO_2_ was well maintained in SnO_2_@C@half-rGO, SnO_2_@C/rGO and SnO_2_/C after the synthesis, as confirmed by XRD patterns (Fig. 3a). The SnO_2_ grains in SnO_2_/C possess the largest size (6.00 nm, Supplementary Table S1), while the ones in SnO_2_@C@half-rGO and SnO_2_@C/rGO are smaller (4.91 and 4.63 nm, respectively). It is concluded that both SGO and LGO have inhibition effect on the grain growth of the SnO_2_ nanoparticles during the hydrothermal process or calcination. The reduction of GO in SnO_2_@C@half-rGO and SnO_2_@C/rGO was confirmed by Raman spectroscopy (Fig. 3b). Typical *D* band (∼1350 cm^−1^) and *G* band (∼1590 cm^−1^) were observed for all the GO-contained samples. As *G* band is the signal of sp^2^ carbon while *D* band is usually regarded as an indication of the disorder in GO or the existence of amorphous carbon, the intensity ratio of *D* and *G* (denoted as *I*_*D*_ */I*_*G*_) is usually calculated to indicate the extent of reduction of GO^25, 26^. The *I*_*D*_ */I*_*G*_ values of SnO_2_@C@half-rGO and SnO_2_@C/rGO were determined to be 0.89 and 0.82, respectively, which are both lower than those of SGO (0.98) and LGO (0.91), despite the influence of the amorphous carbon in both samples. This decrease of *I*_*D*_ */I*_*G*_ demonstrates the successful reduction of GO, which can lead to high electrical conductivity and benefit the service performances of the composites.

The contents of SnO_2_ in SnO_2_@C@half-rGO and SnO_2_@C/rGO were measured to be 63.6 and 68.5 wt%, respectively (TGA, Supplementary Fig. S6). By the further analysis of the X-ray photoelectron spectroscopy (XPS) results, the mass fraction values of SnO_2_ in SnO_2_@C@half-rGO and SnO_2_@C/rGO were determined as 56.5 and 28.5 wt% (Supplementary Fig. S7, Supplementary Table S2), respectively, which are quite different from the TGA results (Supplementary Table S3). It is known that the photoelectron signal detected in XPS spectra is mostly restricted at the surface of the samples. The distinct different content values of SnO_2_ from TGA and XPS for SnO_2_@C/rGO (68.5 wt% to 28.5 wt%) is related to the wholly-wrapped structure (Supplementary Fig. S2), with SnO_2_ clusters covered with the dual thick carbon shell. On the contrary, the smaller difference analyzed by TGA and XPS (63.6 wt% to 56.5 wt%) for SnO_2_@C@half-rGO is ascribed to a thinner carbon shell, and thus gives another evidence for the formation of a partitioned-pomegranate-like structure.

The specific surface area (SSA) of SnO_2_@C@half-rGO is measured to be 375 m^2^ g^−1^ (Fig. 3c), which is more than twice that of LGO-based SnO_2_@C/rGO (177 m^2^ g^−1^) and three times that of the rGO-free comparison SnO_2_/C (124 m^2^ g^−1^). Compared with SnO_2_/C, the higher specific surface area of the GO-based samples can be interpreted as the dispersive effect of both SGO and LGO on SnO_2_ nanoparticles. However, the LGO sheets (SnO_2_@C/rGO) intend to wholly wrap the SnO_2_ clusters, and thus result in the less value than that of SnO_2_@C@half-rGO. The pore size is centered at ∼9 nm for all the three samples, derived from P123 during calcination process (Fig. 3d)^27^. The delicate nano-structure of SnO_2_@C@half-rGO along with the ultra-small size of the SnO_2_ nanoparticles is expected to afford remarkable lithium storage performance.

**Figure 4:**
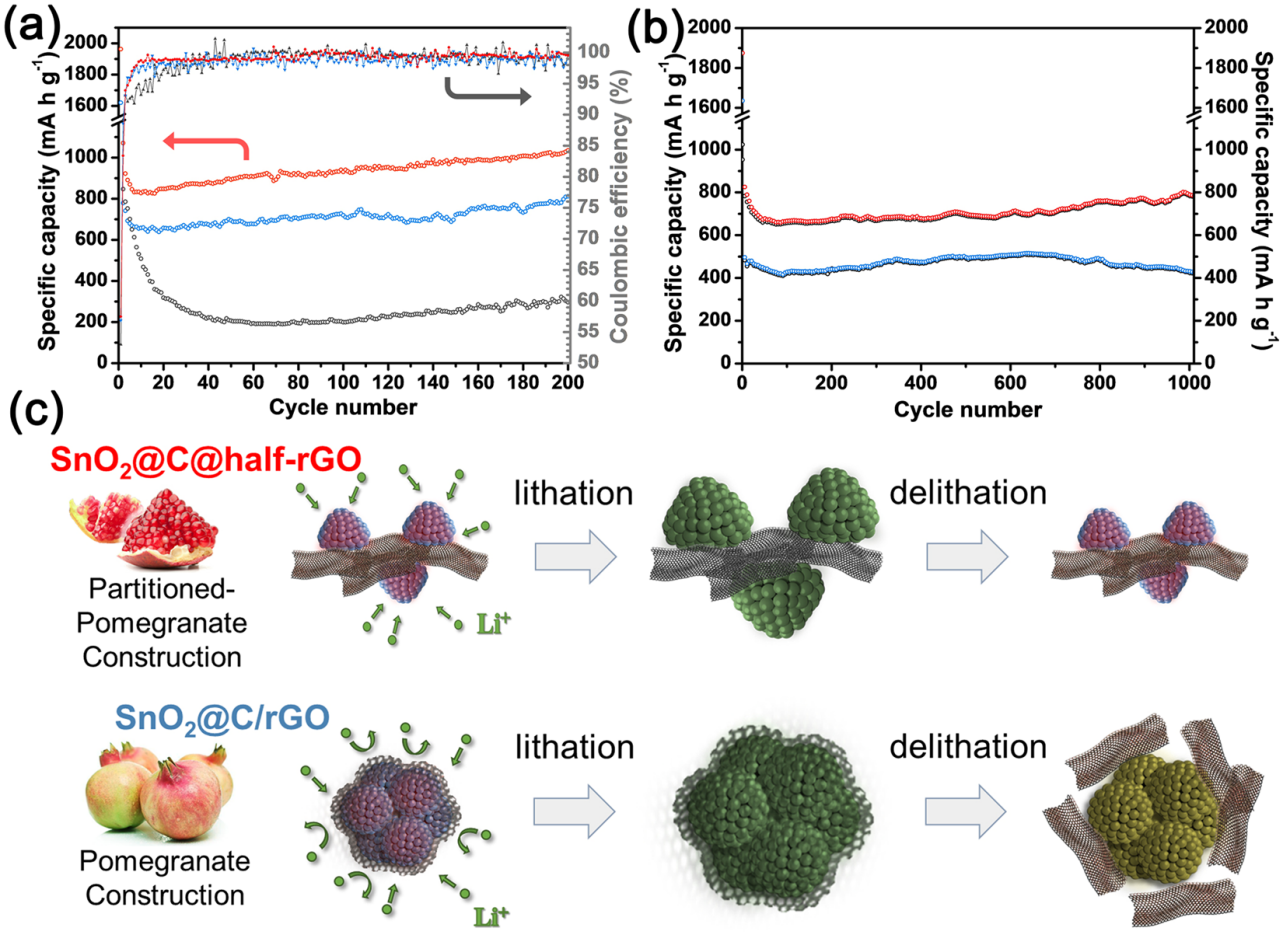
(**a**) Specific discharge capacity and coulombic efficiency of SnO_2_@C@half-rGO (red), SnO_2_@C/rGO (blue) and SnO_2_/C (grey) at 100 mA g^−1^. (**b**) Specific charge and discharge capacity of SnO_2_@C@half-rGO (red) and SnO_2_@C/rGO (blue) at 1 A g^−1^. (**c**) Mechanism of the structure evolution of SnO_2_@C@half-rGO and SnO_2_@C/rGO during lithiation/delithiation.

Galvanostatic charge−discharge tests were first conducted at the low current density of 100 mA g^−1^ (Fig. 4a). In contrast to the fast capacity fading of SnO_2_/C, only a slight declination was observed for SnO_2_@C@half-rGO and SnO_2_@C/rGO in the first few cycles with high capacities maintained. After 40 cycles, the discharge capacities of SnO_2_@C@half-rGO and SnO_2_@C/rGO were 879.6 mAh g^−1^ and 674.0 mAh g^−1^, respectively. Whereas, only 221.9 mAh g^−1^ was remained for the control sample SnO_2_/C. In order to find the reason why SnO_2_@C@half-rGO has a higher capacity than SnO_2_@C/rGO and SnO_2_/C, the cyclic voltammograms (CVs) of SnO_2_@C@half-rGO, SnO_2_@C/rGO and SnO_2_/C for the first three cycles were recorded (Supplementary Fig. S8). The alloying/dealloying reaction of Li_x_Sn species (corresponding to the anodic peaks at 0.1 V and cathodic peaks at 0.5 V) is similar for all the three samples. However, the conversion reactions of SnO_2_ and Sn are quite different. For SnO_2_@C@half-rGO, the anodic peak at 0.9 V (SnO_2_ converting into Sn) in the first sweep process is more apparent than that for SnO_2_@C/rGO and SnO_2_/C. There are two cathodic peaks (at 1.2 V and 1.8 V) for SnO_2_@C@half-rGO, indicating the reversible conversion of Sn back to SnO and further SnO_2_, and two corresponding anodic peak (at 0.9 V and 1.1 V) in the subsequent sweep process can be found. This reversible reaction has been deduced to be possible when the SnO_2_ particles are of nano-size^28^, and is contributive for the excess capacity of SnO_2_@C@half-rGO. For SnO_2_@C/rGO and SnO_2_/C, the weak and single peaks at 1.2 V (cathodic) and 0.9 V (anodic) reveal the less reversibility of the conversion reaction of SnO_2_ and Sn.

Interestingly, after tested for 200 cycles at 100 mA g^−1^, the discharge capacity of SnO_2_@C@half-rGO reached 1034.5 mAh g^−1^, while those of SnO_2_@C/rGO and SnO_2_/C reached 811.9 mAh g^−1^ and 296.3 mAh g^−1^, respectively. To determine the reason for this capacity recovery phenomenon, TEM image of SnO_2_@C@half-rGO after tests was taken as shown in Supplementary Fig. S9. Obviously, the porous cluster structure of SnO_2_ nanoparticles was preserved very well after 200 cycles. In coincidence with the up-to-date reported fundamental research of SnO_2_ anodes^29^, the SnO_2_ phase all changed to tetragonal Sn after 200 cycles, which is confirmed in SAED (Supplementary Fig. S9b inset) and XRD patterns (Supplementary Fig. S10). The Sn nanoparticles were observed to possess more separated and loose distribution on rGO sheets (Supplementary Fig. S9) than in the original morphology of SnO_2_@C@half-rGO (Fig. 2), which may be derived from the relaxation caused by repeated expansion-shrinkage process of Sn. Because of the fine particle size and the protective carbon shell, the Sn nanoparticles stay in adherent during the lithiation/delithiation reaction. Thus the incremental interface between Sn and amorphous carbon may provide more reaction sites for Li_2_O reversibly delithiating and lithiating, which contributes the increasing capacity^29^.

When tested at a high current density of 1 A g^−1^ (Fig. 4b), SnO_2_@C@half-rGO kept increasing for over 1000 cycles and reached 794.7 mAh g^−1^ at 1000 cycles. While the capacity of SnO_2_@C/rGO began to fade after 650 cycles and only maintained 431.8 mAh g^−1^ after 1000 cycles. The structure evolution mechanism of SnO_2_@C@half-rGO and SnO_2_@C/rGO during lithiation/delithiation is schematically shown in Fig. 4c. The wholly-wrapped structure in SnO_2_@C/rGO cannot bear cycling at such a high current density for hundreds of times, probably because the inadequate pore volume of the SnO_2_ clusters along with the severe volume change may have the rGO shell split. On the other hand, the half-supported structure of SnO_2_@C@half-rGO, like partitioned pomegranates, is not only beneficial to the swift Li^+^ transfer at high current density, but also capable to sustain the integrity of the rGO matrix for a long run.

**Figure 5:**
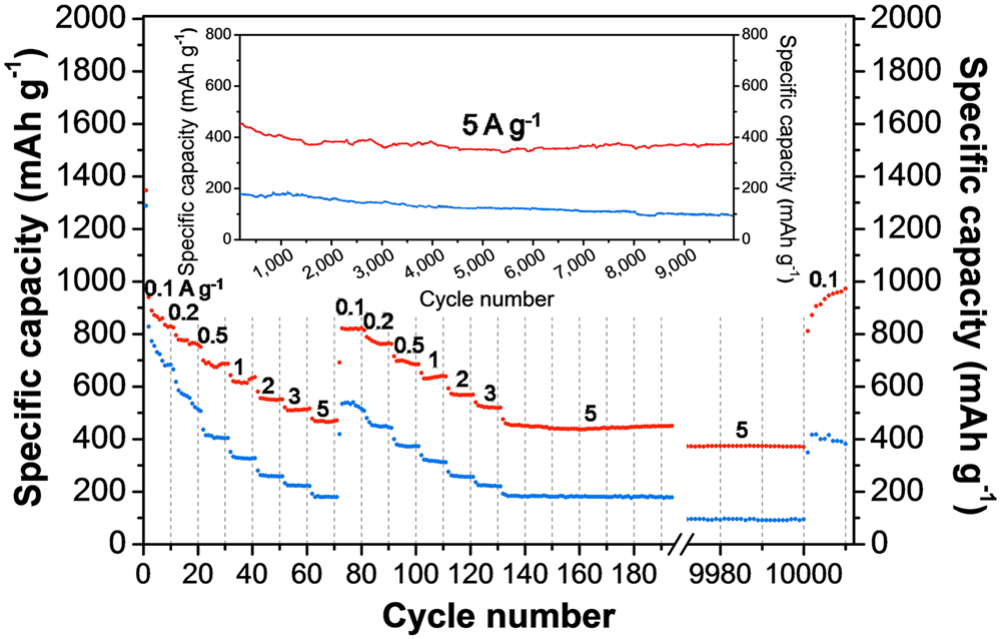
Rate performance (discharge capacities) of SnO_2_@C@half-rGO (red) and SnO_2_@C/rGO (blue). The varying current is 100, 200, 500, 1000, 2000, 3000 and 5000 mA g^−1^ for 10 cycles each, following 100–3000 mA g^−1^ for 10 cycles each again, and 5 A g^−1^ for 9870 cycles until the final 100 mA g^−1^ for 10 cycles.

The rate performance of SnO_2_@C@half-rGO and SnO_2_@C/rGO tested at stepped-incremental current densities is displayed in Fig. 5. In the first round of incremental current densities from 100 mA g^−1^ to 5 A g^−1^ throughout the first 70 cycles, the capacities of SnO_2_@C/rGO decreased much faster than those of SnO_2_@C@half-rGO. Only 179.7 mAh g^−1^ remained for SnO_2_@C/rGO at 5 A g^−1^ (the 70^th^ cycle), while for SnO_2_@C@half-rGO at same condition 469.1 mAh g^−1^ remained. In the subsequent second round from the 71^st^ to 140^th^ cycle, the capacities of SnO_2_@C@half-rGO recovered to 821.1 mAh g^−1^ fast at 100 mA g^−1^ (the 72^nd^ cycle), which was comparable to those at 100 mA g^−1^ in the first round (824.9 mAh g^−1^ for the 10^th^ cycle). As for SnO_2_@C/rGO, the capacity of the 72^nd^ cycle was only 534.0 mAh g^−1^, far smaller than that of the 10^th^ cycle (665.0 mAh g^−1^), which means a big loss of lithium-storage capability after the preceding cycles. The weakness of the wholly-wrapped structure was evident, as the rGO-encapsulated structure of SnO_2_@C/rGO can seal off the mesopores inside the SnO_2_ clusters and thus is not beneficial to the rapid transfer of Li^+^, especially at high current densities. A long-period test at a high current density (5 A g^−1^) was carried out after the previous two round of rate test. The capacities of SnO_2_@C@half-rGO decreased to 370.3 mAh g^−1^ for the 10000^th^ cycle, which is still comparable to the theoretical capacity of the conventional graphite anode for LIBs. Finally, the current density was tuned back to 100 mA g^−1^, and a gradual capacity recover process of SnO_2_@C@half-rGO was recorded. 972.3 mAh g^−1^ was reached in the final cycle, even higher than that of the 72^nd^ cycle. This phenomenon can be ascribed to the activation effect of the very high current density^30^. It is worth mentioning that the high capacities, long service durability and satisfactory rate performance of SnO_2_@C@half-rGO are superior to most of reported SnO_2_-rGO composite anodes with hierarchical structures (shown in Supplementary Table S4).

Encouraged by the excellent rate performance and the stability at high current densities of SnO_2_@C@half-rGO as anode for LIBs, we can reasonably expect its good performance as an anode for LIHCs. It is necessary to mention that SnO_2_ is essentially not a good candidate for LIHCs, because lithiation/delithiation of bulk SnO_2_ occurs in-depth rather than near-surface, which seems inconsistent with the demand for high power density of supercapacitors. However, as for SnO_2_@C@half-rGO here reported, the ultra-small size of the SnO_2_ nanoparticles, the ultimate conductivity of rGO, and the exposed mesopores among the SnO_2_ clusters have changed the intrinsic properties of SnO_2_ and make it a suitable anode for LIHCs.

Here, we chose the activated carbon with porous nature and graphene-like layers (gpC) as the cathode (Supplementary Fig. S11), which is prepared according to our previous report^31^. The prelithiated anodes were coupled with gpC cathode into complete LIHCs (SnO_2_@C@half-rGO//gpC or SnO_2_@C/rGO//gpC).

**Figure 6:**
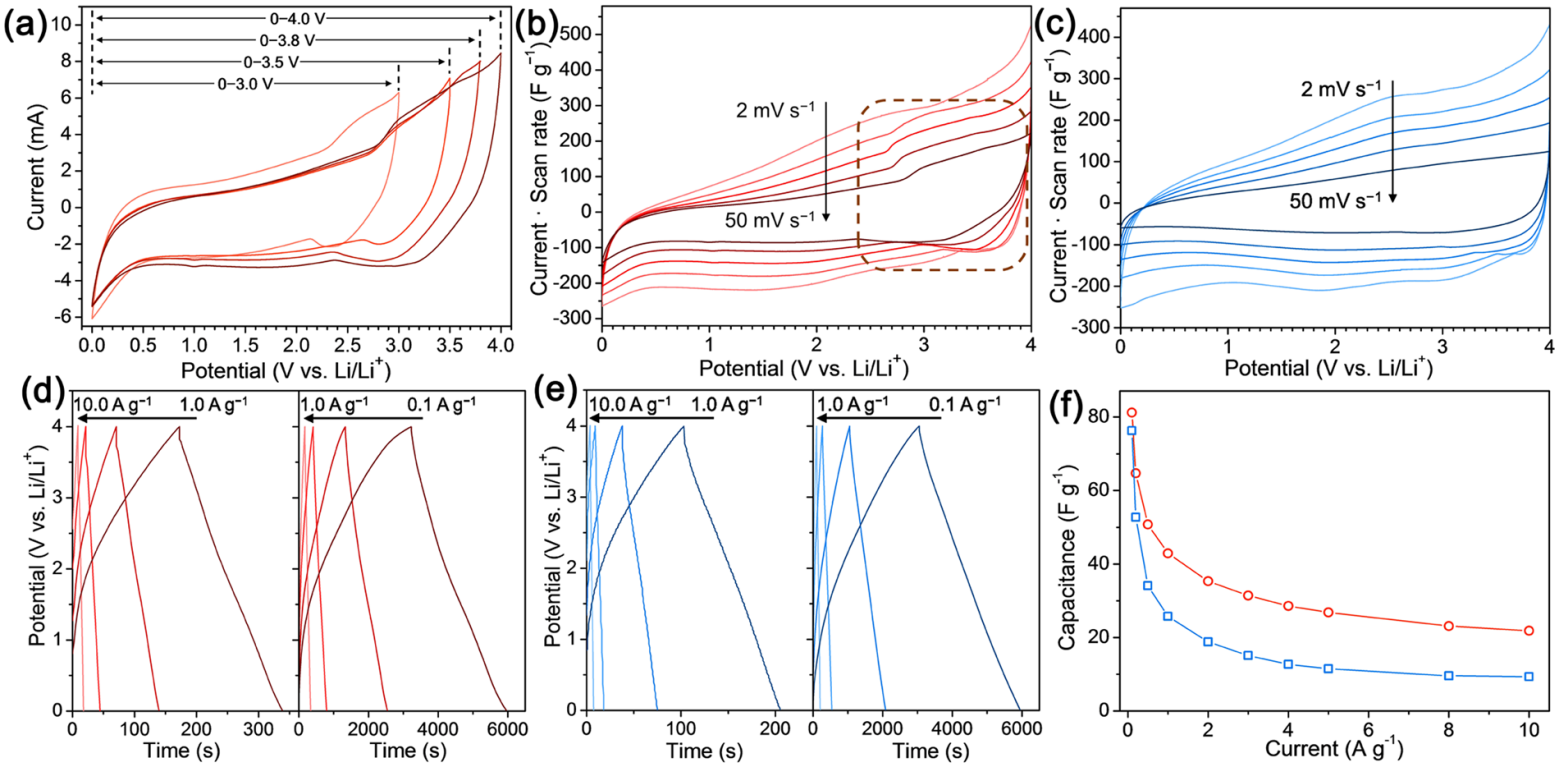
(**a**) The CV curves of SnO_2_@C@half-rGO//gpC LIHC in different voltage windows of 0–3.0 V, 0–3.5 V, 0–3.8 V and 0–4.0 V at 50 mV s^−1^. The CV curves of (**b**) SnO_2_@C@half-rGO//gpC and (**c**) SnO_2_@C/rGO//gpC LIHC at different scan rates of 2, 5, 10, 20, 50 mV s^−1^ in the voltage windows of 0–4.0 V. Galvanostatic charge-discharge curves of (**d**) SnO_2_@C@half-rGO//gpC and (**e**) SnO_2_@C/rGO//gpC LIHC at different current densities of 0.1, 0.2, 0.5, 1.0, 2.0, 5.0, and 10.0 A g^−1^. (**f**) Specific capacitance values of SnO_2_@C@half-rGO//gpC (red) and SnO_2_@C/rGO//gpC (blue) LIHC calculated from the result shown in (**d,e**).

The CV curves of SnO_2_@C@half-rGO//gpC LIHC in different voltage windows of 0–3.0 V, 0–3.5 V, 0–3.8 V and 0–4.0 V all show a similar shape (Fig. 6a), indicating its capability to serve over the voltage window up to 4 V. It is acknowledged that with a same capacitance (*C*) value, the energy density (*W*) increases quadratically with the growth of voltage (*V*), according to the following formula:

1 $$W=1/2\cdot C\cdot {V}^{2}$$

Therefore, the voltage window of 0–4 V was chosen so that high energy densities can be achieved. Different from the symmetric rectangular CV curves of EDLCs, the CV curves of SnO_2_@C@half-rGO//gpC LIHC (Fig. 6b) possess slightly asymmetric and humped shapes. The broad peaks between 2.5–4.0 V on both the anodic and cathodic curves (marked area) keep their presence with the scan rate increasing, which manifests that the faradaic pseudocapacitance of SnO_2_ in SnO_2_@C@half-rGO can be adequately exerted. In contrast, the control sample SnO_2_@C/rGO//gpC showed quite different properties (Fig. 6c). As the scan rate rises, the aforementioned broad peaks between 2.5–4.0 V gradually fade away. The peak-less shape of the CV indicates an EDLC-like property of SnO_2_@C/rGO. It is deduced that the wholly-wrapped structure of SnO_2_@C/rGO would impede the transfer of Li^+^ and hinder the capacitive performance from the SnO_2_ inside the rGO shell, especially in a fast charge−discharge process.

Figure 6d and e show the charge-discharge curves at different current densities for SnO_2_@C@half-rGO//gpC and SnO_2_@C/rGO//gpC LIHCs. Both the curves exhibit quasi-triangular shape. The specific capacitance values were calculated based on the sum mass of active materials on anodes and cathodes and are presented in Fig. 6 f. The capacitance of SnO_2_@C@half-rGO//gpC is higher than that of SnO_2_@C/rGO//gpC at each current density from 0.1–10.0 A g^−1^.

**Figure 7:**
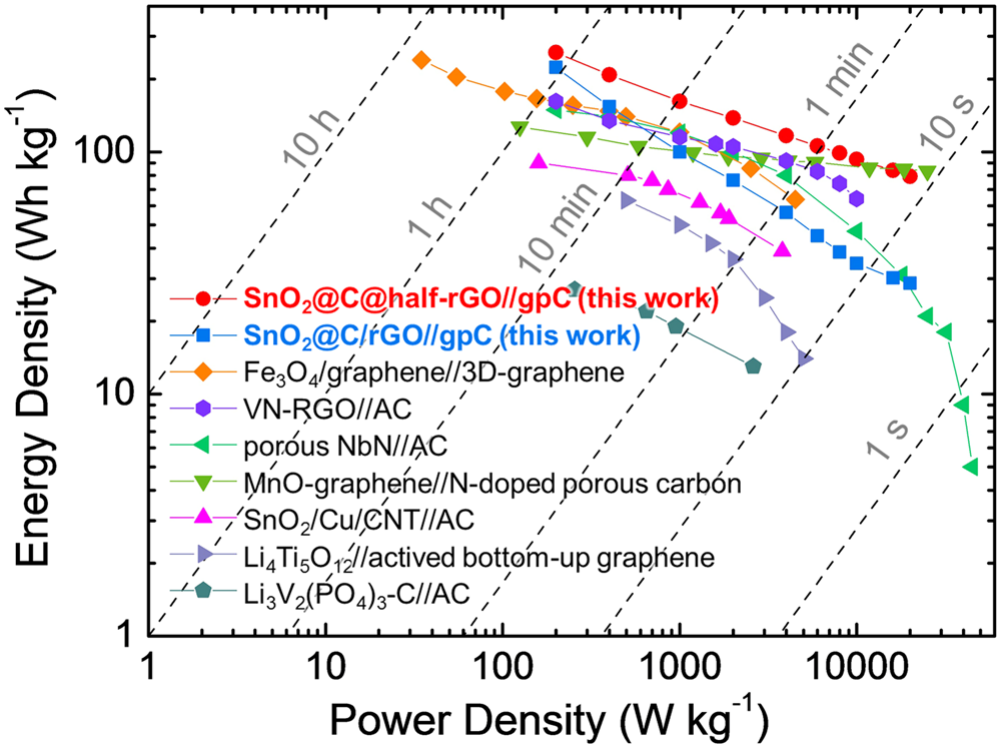
Ragone chart of SnO_2_@C@half-rGO//gpC and SnO_2_@C/rGO//gpC. The performance is compared with reported similar LIHC systems including Fe_3_O_4_/graphene//3D-graphene^32^, VN-RGO//AC^33^, porous NbN//AC^34^, MnO@graphene//HNC^35^, SnO_2_/Cu/CNT//AC^36^, Li_4_Ti_5_O_12_//activated bottom-up graphene^37^, and Li_3_V_2_(PO_4_)_3_-C//AC^38^.

The energy density of SnO_2_@C@half-rGO//gpC at the average power density of ∼200 W kg^−1^ was determined to be 257 Wh kg^−1^. Even at a much higher average power density of ∼20 kW kg^−1^, SnO_2_@C@half-rGO//gpC can still maintain an energy density of 79 Wh kg^−1^. The values of the specific energy vs. specific power from this work are used to plot a Ragone chart (Fig. 7), in comparison with literature results of similar LIHC systems^32–38^. The dominant energy densities of both SnO_2_@C@half-rGO//gpC and SnO_2_@C/rGO//gpC over other LIHC systems at low power densities are attributed to the ultra-small size of the SnO_2_ nanoparticles and the mesopores between them, which create a large surface area for the sufficient redox reaction between SnO_2_ and Li^+^. Along with the increased power densities, the energy densities of SnO_2_@C@half-rGO//gpC maintain the superiority over other reported LIHC systems. It is easy to make out that at relatively high power densities there is more difference between the energy densities of SnO_2_@C@half-rGO//gpC and SnO_2_@C/rGO//gpC, which demonstrates the superiority of the half-open structure of SnO_2_@C@half-rGO. Although both SnO_2_@C@half-rGO//gpC and SnO_2_@C/rGO//gpC benefit from the mesopores inside the SnO_2_ clusters and the conductive rGO matrix outside, the half-open structure gives SnO_2_@C@half-rGO a larger exposed surface than SnO_2_@C/rGO for the high-speed transfer of Li^+^.

**Figure 8:**
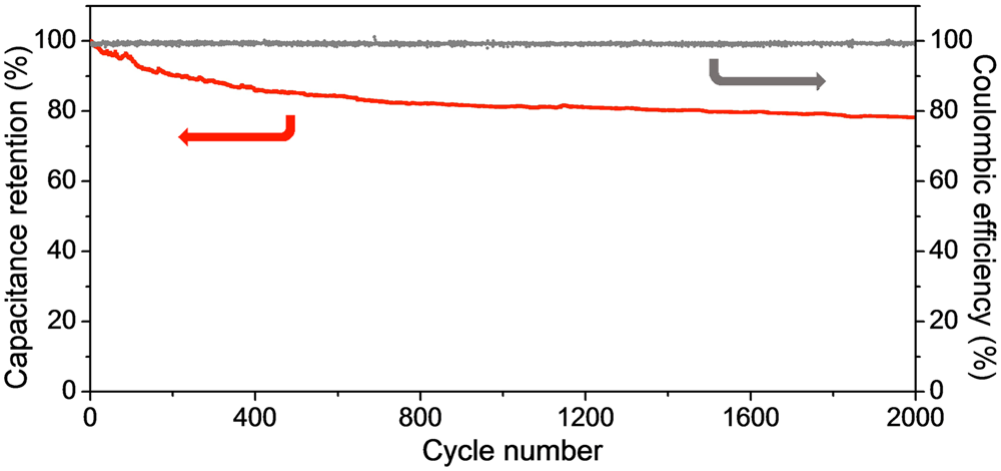
The capacitance retention and coulombic efficiency of SnO_2_@C@half-rGO//gpC LIHC for 2000 cycles at 1.0 A g^−1^.

Moreover, SnO_2_@C@half-rGO//gpC also held the coulombic efficiency nearly 100% throughout the 2000 cycles and exhibited excellent cycle stability (Fig. 8). The capacitance retention was up to 78.2% after 2000 cycles at 1.0 A g^−1^, much higher than the corresponding value of SnO_2_@C/rGO//gpC (56.5%, Supplementary Fig. S12).

Our partitioned-pomegranate-structured materials are different from previously reported pomegranate-like particles that have a closed outer and inner surface. The partially opened outer surface of our material will ensure an unimpeded transfer of Li^+^ into the porous clusters, and at the same time the electrolyte-blocking amorphous carbon layer (like the pulp on pomegranate seeds) could prevent the SEI formation to the surface of the secondary particle. Owing to this structure, it is not surprised that the anode affords such a high cycling performance, especially high rate performance for LIBs, as well as superior performance as anodes for LIHCs.

## Discussion

Inspired by nature, we designed a partitioned-pomegranate-like structure anode material which showed an enhanced lithium storage performance. The porous and micro-sized clusters of SnO_2_ nanoparticles are partially supported by small sheets of reduced graphene oxide. The mesopores inside the clusters remain open to electrolyte for the rapid transfer of Li^+^, which is superior to the wholly-wrapped structure originated from using large sheets of graphene oxide. The synthesized materials maintain a high reversible capacity (1034.5 mAh g^−1^ at 100 mA g^−1^ after 200 cycles), superior rate performance and excellent durability (370.3 mAh g^−1^ after 10000 cycles at 5 A g^−1^). When assembled as anode for LIHCs, a high energy density up to 257 Wh kg^−1^ at ∼200 W kg^−1^ was obtained. Even at a very high power density up to ∼20 kW kg^−1^, the LIHC can still afford 79 Wh kg^−1^. The result initiates a new architecture for graphene-based composite anode materials for LIBs and LIHCs, which is promising to be applied on other new-type anode materials. Furthermore, the performance of LIHCs reported herein is limited by the intrinsic electrochemical property of SnO_2_, and therefore, it is prospectively feasible to apply this graphene-half-supported structure to other materials such as MnO_2_ and RuO_2_, which are more suitable for pseudocapacitors.

## Methods

### Synthesis of SnO_2_ nanoparticles

The SnO_2_ nanoparticles were synthesized as colloidal dispersion. Typically, ammonia aqueous solution (25 wt%, 20 mL) was added dropwise into aqueous solution of SnCl_4_ under severe stirring. The precipitate was collected by centrifugation and washed with deionized water for several times until its pH was lower than 9.0. The washed precipitate was dispersed in deionized water (50 mL) again with sufficient stirring overnight, and the colloidal dispersion of SnO_2_ nanoparticles was thus obtained.

### Synthesis of small sheets of graphene oxide (SGO)

The small sheets of graphene oxide was prepared by ultrasonically shredding the large sheets of graphene oxide (LGO), which was prepared in advance by modified Hummers method using flake graphite (325 mesh) as raw material^39, 40^. LGO aqueous dispension (2 mg mL^−1^, 500 mL) was tuned to alkalescent (pH = 10.0) and then treated by high-power ultrasonication for 2 h. Hydrochloric acid was dropped into the treated dispersion to lead to flocculation of the shredded graphene oxide, which was then collected by centrifugation and cleaned by dialysis. SGO was thus obtained and reserved as aqueous dispersion with concentration determined to be 5.0 mg mL^−1^.

### Synthesis of SnO_2_@C@half-rGO

Pluronic P123 was introduced as surfactant to trigger the formation of SnO_2_ clusters^12^. Typically, 0.1 g of P123 was mixed with the as-prepared colloidal dispersion of SnO_2_ nanoparticles (40 mg mL^−1^, 3 mL), and after complete dissolution of P123, glucose (0.1 g, dissolved in 3 mL of deionized water) and the as-prepared SGO dispersion (4 mL) was added with sufficient stirring. After transferring the mixture into a polytetrafluoroethylene autoclave, kept at 180 °C for 3 h, the resultant sponge of SnO_2_-SGO was freeze-dried. The subsequent fumigation with hydrazine hydrate at 120 °C for 2 h^41^ and heat treatment at 500 °C for 2 h in nitrogen led to the reduction of SGO and the full carbonization of glucose derivatives, and SnO_2_@C@half-rGO was thus formed. For the synthesis of wholly-wrapped clusters of SnO_2_ nanoparticles (SnO_2_@C/rGO), same route were executed except LGO was utilized instead of SGO. Another control sample was prepared with the same route except that no GO was not introduced, and the product only contains SnO_2_ and amorphous carbon, which is simply named as SnO_2_/C.

### Synthesis of graphitized porous carbon (gpC)

Rice husk was carbonized at 650 °C and then activated by KOH at 750 °C with a mass ratio of 1:1. The activated carbon was immersed in Fe(NO_3_)_3_ aqueous solution (0.2 mol L^−1^) with severe sonication treatment for 10 min. After drying at 80 °C overnight, the Fe(NO_3_)_3_/activated carbon mixture were graphitized at 800 °C for 3 h. The ferric impurity was washed by chlorhydric acid (5 wt%), and gpC was thus obtained.

### Materials Characterization

XRD patterns were recorded on a Rigaku D/max2550VL/PC system, using Cu *K*α radiation. SEM (JEOL JSM-6360LV, 15 kV), TEM and HRTEM (JEOL 2010, 200 kV) were used to observe the morphology of the samples. Nitrogen adsorption measurements were performed at 77 K on a Micromeritics ASAP 2020. BET specific surface area was calculated using the *P*/*P*_0_ data between 0.10 and 0.25. Raman spectra were collected on a Renishaw in Via Raman microscope. TGA was conducted on a Perkin-Elmer TGA-7 Thermal Analyzer under O_2_ flow, with a heating rate of 20 °C min^−1^. XPS spactra were recorded on a Perkin-Elmer PHI-5400 spectrometer, using Mg *K*α radiation as the excitation source.

### Electrochemical Measurements

For half cell tests, all the samples (SnO_2_@C@half-rGO, SnO_2_@C/rGO and SnO_2_/C) were assembled into 2016 coin cells with Li^0^ foil as counter electrodes. To prepare working electrode, the samples were mixed with acetylene black and PVDF by a mass ratio of 8:1:1, and then coated on a copper foil with a mass/area ratio of ∼2 mg cm^−2^. Polypropylene membrane (Celgard 2500) was employed as separator. LiPF_6_ (1 mol L^−1^) in ethylene carbonate (EC) and diethyl carbonate (DEC) (1:1, v/v) was used as electrolyte. All the cells were assembled in inert atmosphere and tested at room temperature. CV tests were done on a CHI-660D electrochemical workstation. Galvanostatic cycling tests were conducted on a LAND-CT2001A battery test system, with a voltage window of 0.01–3.00 V vs. Li/Li^+^. For LIHCs, the samples were first coated on copper foil and assembled in to coin cells with Li^0^ foil following the same process as above, and discharged to 0 V at a current density of 100 mA g^−1^. The prelithiated anodes were taken out and washed with DEC for three times. The gpC was mixed with acetylene black and PVDF by a mass ratio of 8:1:1, coated on aluminium foil to be cathode, and then assembled into coin cells with the prelithiated anodes. The mass ratio of cathode/anode in the LIHC devices was 3:1.

## Electronic supplementary material

Supplementary Information
