# Supplementary material for: Construction of SnO2−Graphene Composite with Half-Supported Cluster Structure as Anode toward Superior Lithium Storage Properties
Source: Sci Rep. 2017 Jun 12;7:3276. doi: 10.1038/s41598-017-03603-1 (PMC5468241; doi:10.1038/s41598-017-03603-1)
Supplement: Supplementary file 1 — Supplementary Information [file 41598_2017_3603_MOESM1_ESM.pdf]

Supplementary Information

Construction of SnO_2–Graphene Composite with Half-Supported Cluster Structure as Anode toward Superior Lithium Storage Properties

Chengling Zhu[1], Zhixin Chen[2], Shenmin Zhu[1,3,*], Yao Li[1], Hui Pan[1], Xin Meng[1], Muhammad Imtiaz[1], and Di Zhang[1]

[1]State Key Laboratory of Metal Matrix Composites, Shanghai Jiao Tong University, Shanghai 200240, P. R. China
[2]School of Mechanical, Materials & Mechatronics Engineering, University of Wollongong, Wollongong, NSW 2522, Australia
[3]National Engineering Research Center for Nanotechnology, Shanghai, P. R. China
*smzhu@sjtu.edu.cn

In this research, SGO was prepared by shredding LGO under sonication in alkaline environment. The polarized optical images of the GO dispersion before (LGO) and after the treatment (SGO) are shown in Figure S2. The LGO dispersion displays obvious birefringence, indicating its nematic phase, while no liquid crystalline behavior can be found for SGO. This change in optical property has been verified as a sign of GO's scale decreasing[1], which can be demonstrated more directly by AFM (atomic force microscope) images of LGO and SGO (Figure S3). The mean lateral size of SGO was determined to be 0.42 μm, much smaller than that of LGO (1.65 μm).

The morphology and structures of gpC can be well observed from SEM and TEM images (Figure S11a,b), while the graphitized lattice is clearly shown in Figure S11c. A high specific surface area of 1467 $m^2\ g^{-1}$ was measured with the average pore size of 3.9 nm (Figure S11d). The CV curves of gpC electrode were recorded in the potential window of 3.0–4.5 V (vs Li/Li^+), with Li^0 as the counter electrode (Figure S11e). The symmetric, peak-less and quasi-rectangular shape indicates a typical electrostatic double layer capacitance (EDLC), which was also verified by the almost straight charging/discharging profiles in the subsequent galvanostatic tests (Figure S11f). A high capacitance of 178.3 F g^{-1} was revealed at 0.5 A g^{-1}, while 137.7 F g^{-1} can be maintained at a higher current density of 5 A g^{-1}. The excellent PF_6^--storage performance of gpC allows a good matching with SnO_2@C@half-rGO in LIHC devices.

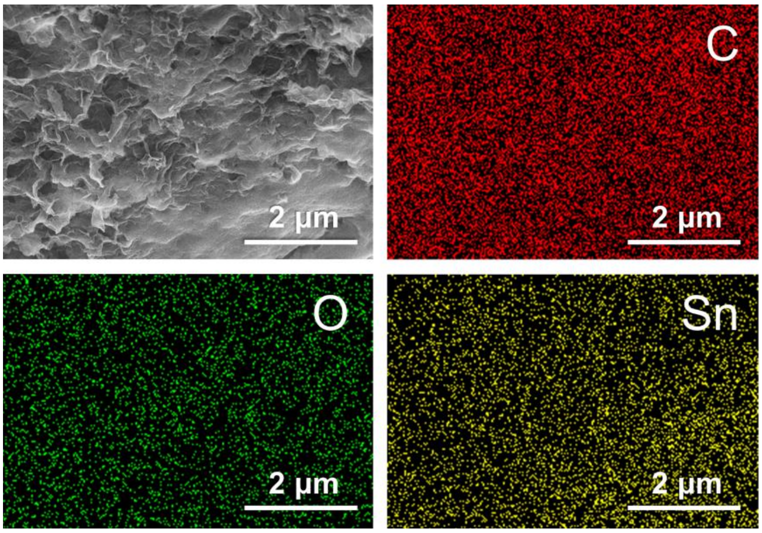

Figure S1. SEM image and the corresponding Energy-dispersive X-ray elemental mapping result of SnO_2@C@half-rGO.

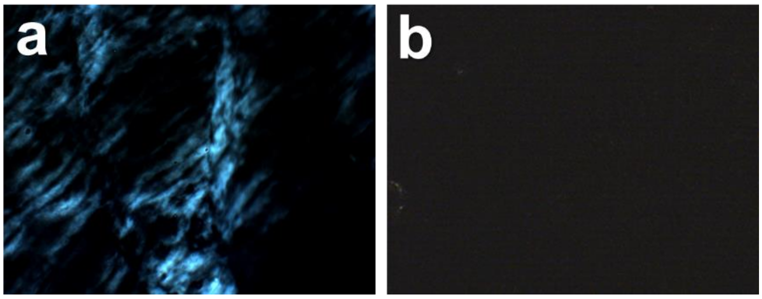

Figure S2. The images of (a) LGO and (b) SGO dispersion of 2 mg mL^{-1} under polarized light microscopy.

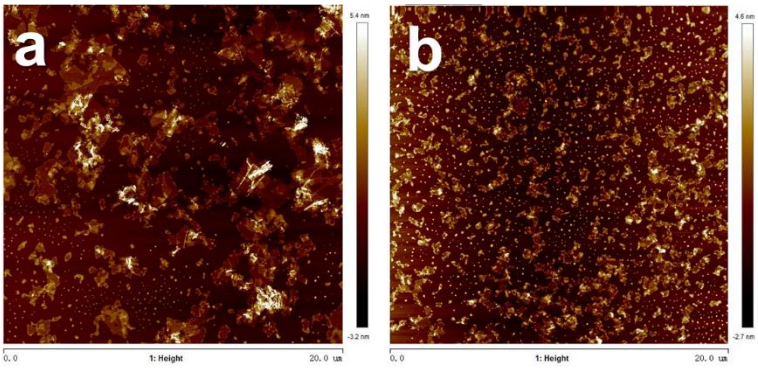

Figure S3. The images of (a) LGO and (b) SGO sheets under atomic-force microscopy.

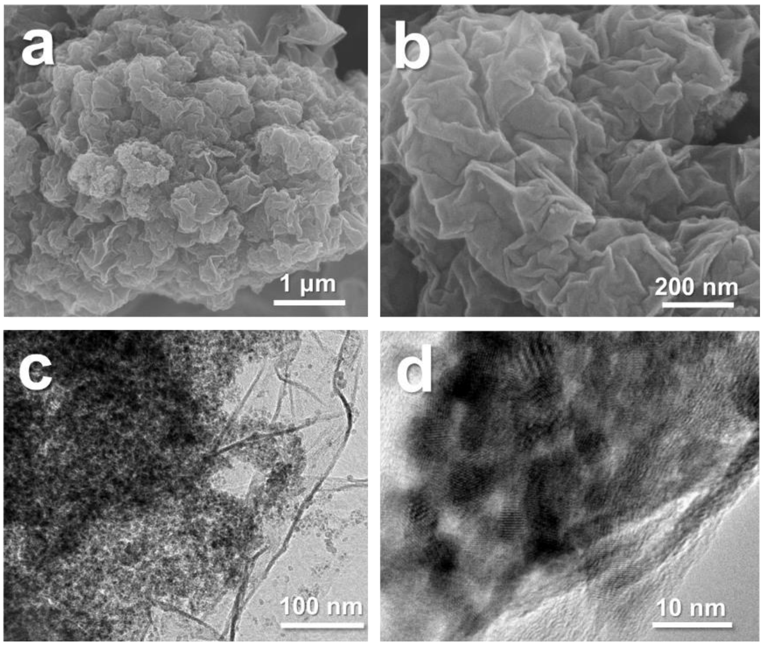

Figure S4. (a,b) SEM and (c,d) TEM images of the LGO-derived sample SnO_2@C/rGO. A wholly-wrapped cluster structure can be well distinguished.

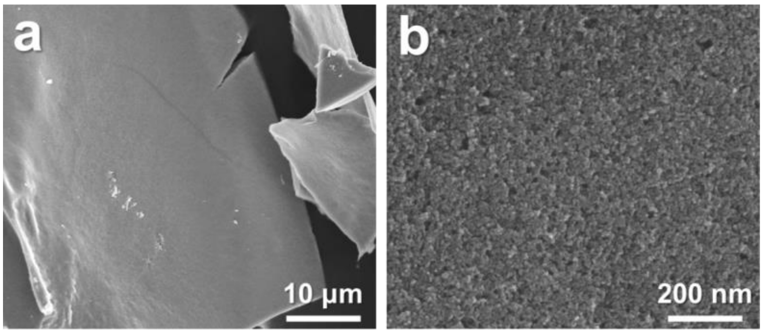

Figure S5. (a,b) SEM images of SnO_2/C, the control sample prepared without GO. A porous surface morphology can be observed in (b).

Table S1. The main grain size of the SnO_2 nanoparticles in the samples, determined by Scherrer equation.

Sample Name	Peak Center	FWHM	Mean Grain Size
SnO_2 nanoparticles	26.61	5.60	1.44
SnO_2@C@half-rGO	26.75	3.29	2.45
SnO_2@C/rGO	26.72	3.49	2.31
SnO_2/C	26.51	2.69	3.00

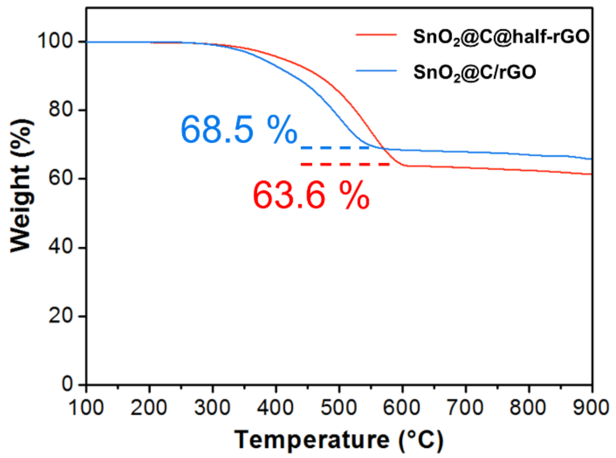

Figure S6. TGA curves of SnO_2@C@half-rGO and SnO_2@C/rGO, with heating rate of 10 ℃ min^{-1} in O_2 atmosphere.

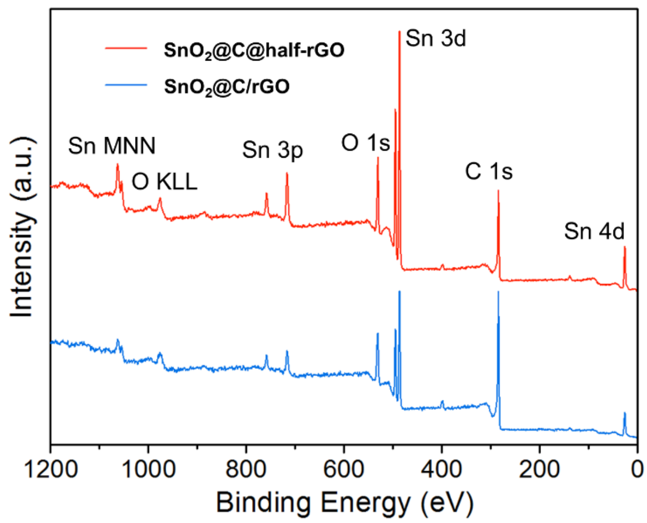

Figure S7. Survey XPS spectra of SnO_2@C@half-rGO and SnO_2@C/rGO.

Table S2. Element contents of C, O, and Sn computed from survey XPS spectra.

Sample Name	Peaks	Position (eV)	FWHM (eV)	Area (T MFP)	Atom Concentration (%)	Mass Concentration (%)
SnO_2@C/rGO	C 1s	284.60	1.28	1.62	81.09	61.46
	O 1s	531.41	3.28	0.32	15.91	16.06
	Sn 3d	487.05	1.40	0.06	3.00	22.47
SnO_2@C@half-rGO	C 1s	284.61	1.12	1.08	66.06	36.52
	O 1s	531.26	1.51	0.42	25.80	19.00
	Sn 3d	487.32	1.20	0.13	8.14	44.48

Table S3. Comparison of the SnO_2 content values in SnO_2@C@half-rGO and SnO_2@C/rGO calculated from TGA and XPS results.

Sample Name	SnO_2 content calculated from TGA results (wt%)	SnO_2 content calculated from XPS results (wt%)[a]
SnO_2@C/rGO	68.5	28.5
SnO_2@C@half-rGO	63.6	56.5

[a]The SnO_2 content values calculated from XPS results are based on the Sn element contents in Table S2, following the equation:

$$w_{\mathrm{SnO_2}} = w_{\mathrm{Sn}} \cdot \frac{M_{\mathrm{SnO_2}}}{M_{\mathrm{Sn}}} = 1.27 w_{\mathrm{Sn}}$$

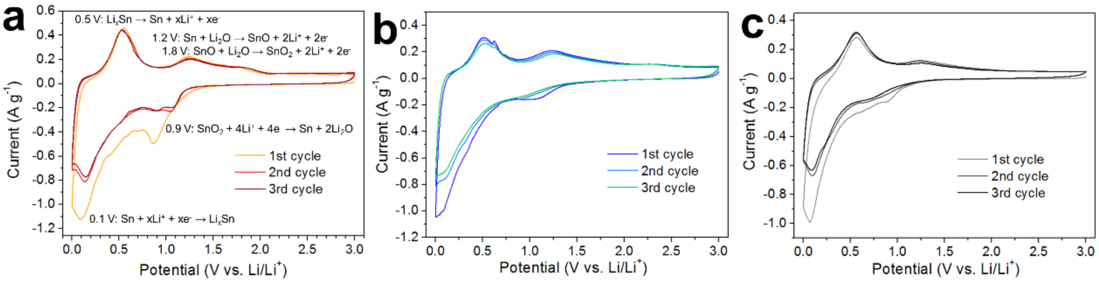

Figure S8. CVs of (a) SnO_2@C@half-rGO, (b) SnO_2@C/rGO and (c) SnO_2/C at 0.1 mV s^{-1} for the first 3 cycles.

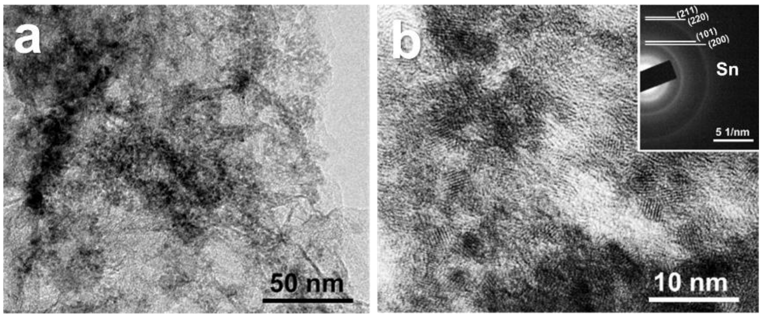

Figure S9. (a,b) TEM images and inset of (b) SEAD pattern of SnO_2@C@half-rGO after tested for 200 cycles at 100 mA g^{-1}.

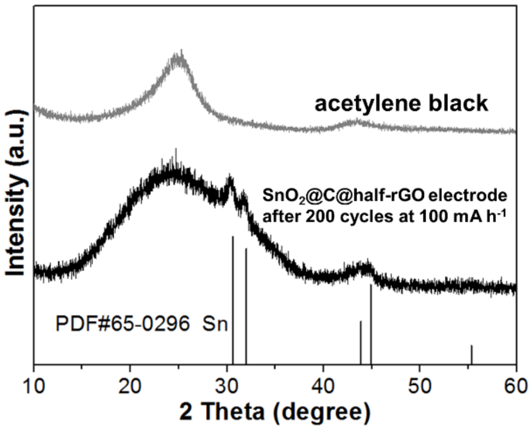

Figure S10. XRD pattern of SnO_2@C@half-rGO after tested for 200 cycles at 100 mA g^{-1}.

Table S4. Compare of the capacities, service durability and rate performance of SnO_2@C@half-rGO with other SnO_2–rGO composite anodes with hierarchical structures reported in literatures.

**SnO_2–rGO composite anodes with hierarchical structures**	**low rate**		**high rate**	
	Capacity (mA h g^{-1}) / after xx cycles	Current density (mA g^{-1})	Capacity (mA h g^{-1}) / after xx cycles	Current density (mA g^{-1})
**SnO_2 nanoparticles fixed on rGO with PANI coating[2]**	~750 / 100	100	~320 / 700	1000
**polydopamine coated rGO/SnO_2 cross-linked with PAA[3]**	718 / 200	100	512 / 50	1000
**SnO_2–rGO nanoribbons[4]**	1027 / 165	100	753 / 600	1000
**graphene-encapsulated hollow SnO_2@SnS_2[5]**	583 / 100	200	487 / 100	500
**Hierarchical TiO_2–SnO_2–graphene aerogels[6]**	750 / 100	100	470 / 150	1000
**3D hierarchical porous SnO_2/graphene frameworks[7]**	830 / 70	100	621 / --	500
**sandwiched graphene/SnO_2nanorod/ carbon nanostructures[8]**	1419 / 150	100	750 / 350	1000
**graphene-based mesoporous SnO_2[9]**	847.5 / 50	78.2	-- / --	--
**flower-like SnO_2 nanoparticles grown on graphene[10]**	658.4 / 50	100	~500 / --	500
**3D carbon-coated SnO_2/rGO foam[11]**	717 / 130	100	-- / --	--
**3D graphene/CNT/SnO_2 hybrid[12]**	842 / 100	200	414 / --	2000
**amorphous ultrathin SnO_2 films on graphene network[13]**	~800 / --	100	410 / 200	1000
**3D Graphene/SWCNT aerogel anchored with SnO_2 nanoparticles[14]**	758 / 200	100	537 / 300	1000
**SnO_2 nanocorals@graphene aerogel[15]**	872 / 50	100	584 / --	1000
**N-doped carbon@SnO_2@Sn/3D graphene-like networks[16]**	901 / 200	100	550 / 500	1000
SnO_2@C@half-rGO in this work	1035 / 200	100	795 / 1000	1000
			370 / 10000	5000

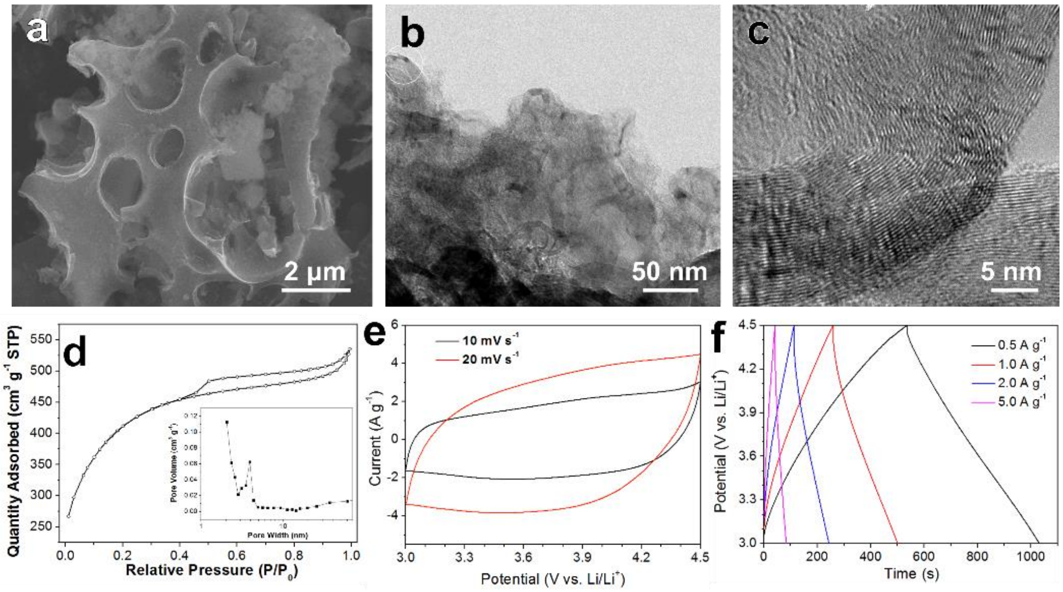

Figure S11. (a) SEM observation of gpC. (b,c) TEM images of gpC with different magnification. (d) Nitrogen adsorption/desorption isotherms and inset pore size distribution of gpC. (e) CV curves of gpC electrode over 3.0−4.5 V vs Li/Li^{+}. (f) Galvanostatic charge-discharge curves of gpC electrode at different current densities of 0.5−5.0 A g^{-1}.

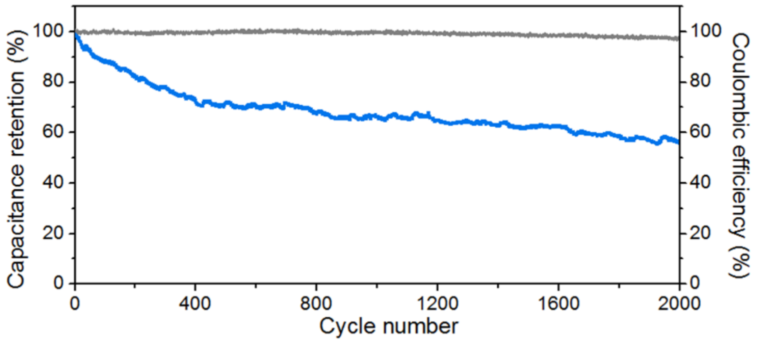

Figure S12. The capacitance retention and coulombic efficiency of SnO_2@C/rGO//gpC LIHC for 2000 cycles at 1 A g^{-1}.

1. Seyedin, M. Z., Razal, J. M., Innis, P. C., Jalili, R. & Wallace, G. G. Achieving Outstanding Mechanical Performance in Reinforced Elastomeric Composite Fibers Using Large Sheets of Graphene Oxide. *Adv. Funct. Mater.* **25**, 94–104 (2015).
2. Dong, Y. *et al.* Dually Fixed SnO_2 Nanoparticles on Graphene Nanosheets by Polyaniline Coating for Superior Lithium Storage. *ACS Appl. Mater. Inter.* **7**, 2444–2451 (2015).

3. Wang, L., Wang, D., Dong, Z., Zhang, F. & Jin, J. Interface Chemistry Engineering for Stable Cycling of Reduced GO/SnO_2 Nanocomposites for Lithium Ion Battery. *Nano Lett.* **13**, 1711–1716 (2013).
4. Li, L., Kovalchuk, A. & Tour, J. M. SnO_2-Reduced Graphene Oxide Nanoribbons as Anodes for Lithium Ion Batteries with Enhanced Cycling Stability. *Nano Res.* **7**, 1319–1326 (2014).
5. Xu, W. *et al.* Hierarchical Graphene-Encapsulated Hollow SnO_2@SnS_2 Nanostructures with Enhanced Lithium Storage Capability. *ACS Appl. Mater. Inter.* **7**, 22533–22541 (2015).
6. Han, S. *et al.* Hierarchical TiO_2–SnO_2–Graphene Aerogels for Enhanced Lithium Storage. *Phys. Chem. Chem. Phys.* **17**, 1580–1584 (2015).
7. Huang, Y. *et al.* Assembly of Tin Oxide/Graphene Nanosheets into 3D Hierarchical Frameworks for High-Performance Lithium Storage. *ChemSusChem* **6**, 1510–1515 (2013).
8. Wang, D. *et al.* Layer by Layer Assembly of Sandwiched Graphene/SnO_2 Nanorod/Carbon Nanostructures with Ultrahigh Lithium Ion Storage Properties. *Energy Environ. Sci.* **6**, 2900–2906 (2013).
9. Yang, S., Yue, W., Zhu, J., Ren, Y. & Yang, X. Graphene-Based Mesoporous SnO_2 with Enhanced Electrochemical Performance for Lithium-Ion Batteries. *Adv. Funct. Mater.* **23**, 3570–3576 (2013).
10. Guo, Q. & Qin, X. Flower-Like SnO_2 Nanoparticles Grown on Graphene as Anode Materials for Lithium-Ion Batteries. *J. Solid State Electrochem.* **18**, 1031–1039 (2014).
11. Tao, H., Zhu, S., Xiong, L., Yang, X. & Zhang, L. Three-Dimensional Carbon-Coated SnO_2/Reduced Graphene Oxide Foam as a Binder-Free Anode for High-Performance Lithium-Ion Batteries. *ChemElectroChem* **3**, 1063–1071 (2016).
12. Zhang, Z., Wang, L., Xiao, J., Xiao, F. & Wang, S. One-Pot Synthesis of Three-Dimensional Graphene/Carbon Nanotube/SnO_2 Hybrid Architectures with Enhanced Lithium Storage Properties. *ACS Appl. Mater. Inter.* **7**, 17963–17968 (2015).
13. Xie, M. *et al.* Amorphous Ultrathin SnO_2 Films by Atomic Layer Deposition on Graphene Network as Highly Stable Anodes for Lithium-Ion Batteries. *ACS Appl. Mater. Inter.* **7**, 27735–27742 (2015).
14. Wang, J. *et al.* Three-Dimensional Graphene/Single-Walled Carbon Nanotube Aerogel Anchored with SnO_2 Nanoparticles for High Performance Lithium Storage. *ACS Appl. Mater. Inter.* **9**, 3544–3553 (2017).
15. Yao, X. *et al.* In Situ Integration of Anisotropic SnO_2 Heterostructures Inside Three-Dimensional Graphene Aerogel for Enhanced Lithium Storage. *ACS Appl. Mater. Inter.* **7**, 26085–26093 (2015).
16. Li, Y. *et al.* Nitrogen-Doped Carbon-Encapsulated SnO_2@Sn Nanoparticles Uniformly Grafted on Three-Dimensional Graphene-like Networks as Anode for High-Performance Lithium-Ion Batteries. *ACS Appl. Mater. Inter.* **8**, 197–207 (2015).
